# Rapid and Accurate Data Processing for Silver Nanoparticle Oxidation in Nano-Impact Electrochemistry

**DOI:** 10.3389/fchem.2021.718000

**Published:** 2021-07-02

**Authors:** Xi-Han Zhao, Yi-Ge Zhou

**Affiliations:** Institute of Chemical Biology and Nanomedicine, College of Chemistry and Chemical Engineering, Hunan University, Changsha, China

**Keywords:** nano-impact electrochemistry, silver nanoparticles, automated data processing, moving average filter, spike detection

## Abstract

In recent years, nano-impact electrochemistry (NIE) has attracted widespread attention as a new electroanalytical approach for the analysis and characterization of single nanoparticles in solution. The accurate analysis of the large volume of the experimental data is of great significance in improving the reliability of this method. Unfortunately, the commonly used data analysis approaches, mainly based on manual processing, are often time-consuming and subjective. Herein, we propose a spike detection algorithm for automatically processing the data from the direct oxidation of sliver nanoparticles (AgNPs) in NIE experiments, including baseline extraction, spike identification and spike area integration. The resulting size distribution of AgNPs is found to agree very well with that from transmission electron microscopy (TEM), showing that the current algorithm is promising for automated analysis of NIE data with high efficiency and accuracy.

## Introduction

Nano-impact electrochemistry (NIE) is a recently developed electroanalytical tool of significant importance that enables the analysis and characterization of single nanoparticles in aqueous solution (Sokolov et al., 2017). In this method, single nanoparticles including but not limited to inorganic nanoparticles (Dasari et al., 2013; Tschulik et al., 2013; Ngamchuea et al., 2017; Zampardi et al., 2017; Li et al., 2018; Peng et al., 2018; Xu et al., 2018), organic nanoparticles (Cheng et al., 2014; Kim et al., 2015; Feng et al., 2016), functional materials (Patrice et al., 2018; Pendergast et al., 2021), and liposomes (Dunevall et al., 2015; Cheng and Compton, 2016; Liu et al., 2018; Lebègue et al., 2020), can stochastically impact on the surface of a microelectrode from Brownian motion. The collision entities are further extended to biospecies, such as enzymes (Lin et al., 2017; Wang et al., 2021), cells (Dick, 2016; Gooding, 2016; Lee et al., 2020), bacterium (Lee et al., 2016; Gao et al., 2018; Chen et al., 2021), and viruses (Sepunaru et al., 2016). During the impact of the single entities to the electrode, electrochemical reactions will take place, including the direct electrolysis of the entities themselves (Zhou et al., 2012a; Zhou et al., 2012b), the electrocatalytic reactions occurring on the surfaces of the entities (Patrice et al., 2018; Xiang et al., 2018; Du et al., 2020),and diffusion blocking of the electroactive species by electrochemically inert entities (Dick et al., 2015; Lee et al., 2019).

The direct electrolysis of nanoparticles was first proposed by the Compton Group in 2011, where single silver nanoparticles (AgNPs) were directly oxidized when colliding to the surface of a carbon fiber microelectrode potentiostatted at an oxidative potential sufficient to oxidize silver (Zhou et al., 2011). In the current-time trace, a spike was referred to a single oxidation event corresponding to AgNPs impact. Assuming that the AgNPs are completely oxidized, the size of single nanoparticles can be obtained by integrating the charge passed per spike based on the Faraday’s Law. Such analytical method was named as anodic particle coulometry (APC), and was extended to the detection and characterization of Au (Zhou et al., 2012b), Ni (Zhou et al., 2013), and Cu (Haddou et al., 2012) nanoparticles. APC was also used to study the aggregation of metal nanoparticles in aqueous solution (Rees et al., 2011; Sokolov et al., 2015) and determine the concentration of nanoparticles (Stuart et al., 2012). Compared to the conventional characterization techniques, such as electron microscopy and dynamic light scattering (DLS), APC allows *in-situ* detection with simplicity, fast response and high throughput. Due to easy engineering and functionalization of the metal nanoparticles, they can be used as electroactive probes for the detection of a variety of biomolecules and bioentities, including proteins (Kirk et al., 2021; Zhang et al., 2021), bacterium (Sepunaru et al., 2015) and viruses (Sepunaru et al., 2016). Since APC based sensors can analyze individual entities, they allow sensitive analysis of the targets, precise counting of bioentities, and better understanding of biological heterogeneity.

**FIGURE 1:**
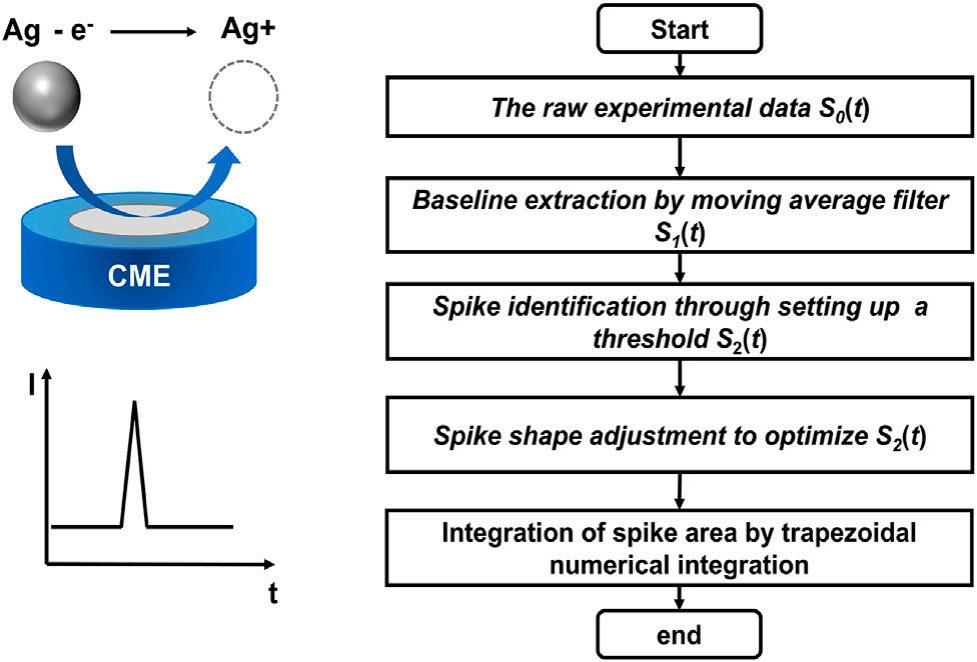
Scheme of the spike detection algorithm for automated data processing of AgNP oxidation in nano-impact electrochemistry. The diagram illustrates the direct oxidation of a AgNP during the impact to the surface of a carbon microelectrode (CME) and the corresponding oxidative spike.

APC based single entity analysis, whether it is a nanoparticle, a bacterial or a cell, all relies on the quantification of the spikes on the current-time traces. Therefore, the accurate recognition and analysis of these spikes play a vital role in improving the reliability of this method. Due to the large data volume and the relatively high background noise compared to the signals (Ma et al., 2017), the commonly used data analysis software products (such as Origin) which require manual processing on the data, are not only time-consuming, but also introduce operator bias to the analysis. Therefore, it is highly desirable to develop automated method for rapid and accurate data processing of APC based analysis. Herein, we demonstrate a spike detection algorithm based on moving average filter and threshold method for automated analysis of APC (Figure 1), enabling fast and accurate processing of a large quantity of data. The current algorithm may provide opportunities for applying NIE into high-throughput sensing applications.

## Materials and Methods

### Chemicals

Silver nitrate, trisodium citrate dehydrate and sodium borohydride were purchased from Sigma Aldrich. Potassium chloride was obtained from Tianjin Guangfu Fine Chemical Research Institute. All solutions were made by using ultrapure water of resistivity ≥18.2 MΩ (Millipore).

### AgNPs Synthesis and Characterization

Citrate-capped AgNPs were synthesized by seed-mediate growth method according to the previous literature (Wan et al., 2013). Briefly, 20 ml of 1% (w/v) citrate solution and 75 ml of water were mixed in a flask and then heated to 70°C for 15 min. Next, 1.7 ml of 1% (w/v) AgNO_3_ solution was introduced to the flask, following the addition of 2 ml of 0.1% (w/v) newly prepared NaBH_4_ solution. The mixture was continuously heated at 70°C with vigorous stirring for 1 h and cooled to room temperature, which was then diluted with water to 100 ml and used as starter seeds. Afterward, 2 ml of 1% citrate solution was added to 80 ml of water in another clean flask and was heated until it was kept boiling for 15 min. Subsequently, 10 ml of the starter seeds solution was introduced in the flask while vigorous stirring, followed by the addition of 1.7 ml of 1% AgNO_3_ solution. The mixture in the flask was kept boiling under stirring before reflux condensation was performed for 1 h. Finally, it was allowed to cool to the room temperature and ready for use.

### Instrumentation

The morphology of AgNPs was characterized by transmission electron microscopy (TEM, JEOL-2010, Japan) supported on a copper film.

### Nano-Impact Electrochemistry

The electrochemical experiments were carried out at room temperature in a three-electrode cell within a double Faraday cage. A carbon fiber microelectrode of 7 μm diameter (ALS Co. Ltd, Japan) was used as a working electrode. Aplatinum wire (XianRen Co. Ltd, Shanghai, China) and a saturated calomel electrode (SCE) (XianRen Co. Ltd, Shanghai, China) were used as a counter electrode and a reference electrode, respectively. The electrochemical measurements were performed on an Autolab PGSTAT 302N from Metrohm-Autolab (BV, Utrecht, Netherlands), fitted with an extremely low-noise (ECD) module to reduce background noise. The sampling rate was 435 Hz (2.3 ms), the current range was set as 0–100 pA, and the default bandwidth (below 100 Hz) was used under such selected current range.

### Data Analysis

The data was processed using a script written by MATLAB R2020a software under Windows 10 with 2.4 GHz Intel Core i5 processors. The amount of data points and spikes being processed were 130,435 and 1,574, respectively, under the selected threshold. By using the function of tic and toc in MATLAB, the time cost for data processing is 15 ± 2 s.

## Results and Discussion

We propose a new simple but effective computational method to detect the current spikes from noisy experimental data. We can then compute numerically the corresponding charge level, allowing the quantification of the size of the nanoparticles. The resulting size distribution from our computation can be matched with that from TEM. The algorithm mainly consists of three computational steps, *i.e.*, baseline extraction, spike identification and spike area integration. The details are discussed below.

### Baseline Extraction

Single AgNPs impact signals were recorded in chronoamperometric profiles where numerous current transients were indicative of single AgNPs oxidation events. Due to the relatively low current intensity produced by single AgNPs, the background noise should be lowered as much as possible in order to better identify and find the valid signals. There already exist many attempts that have been dedicated to improve the signal-to-noise ratio (SNR) of nano-impact measurements, *e.g.,* using a specialized Faraday cage to shield outside disturbance (Bunga and Kataky, 2019), fitting an extremely low-noise module (ECD) into the potentiostat (Bartlett et al., 2015), or implementing a low-pass filtering system (Batchelor-McAuley et al., 2015) to reduce the background noise. However, these methods are not able to fully avoid the interference of the noise, especially in the measurements where small sizes of nanoparticles are analyzed. In addition, the use of low-pass filtering system would very probably cause a distortion to the original signals (Kätelhön et al., 2016; Kanokkanchana et al., 2018).

In order to obtain the electrochemical information of interest from a large number of impact signals, it is necessary to identify these spikes from a noisy background. In the first step, the baseline was extracted by a moving average filter (Gu et al., 2015; Xu et al., 2021), which can minimize the interference of high-frequency noises. As shown in Eq. 1, a moving average of order m can be written as:

$$S_{1}\left(t\right)=\frac{1}{m}\overset{j=k}{\underset{j=-k}{\sum }}S_{0}\left(t+j\right)$$ (1)

where *m* = 2*k*+1, is also known as the length of the averaging window. *S*_*0*_(*t*) is the measured current value at time *t*, whereas *S*_1_(*t*) denotes the baseline signal. For those values at the beginning and at the end, *k* is usually set as the number of available points, *i.e.*, *k* = 0 for *t* = 0, *k* = 1 for *t* = 1, *etc.*

### Spike Identification

Generally, each spike is composed of a cluster of data points above the baseline. Therefore, to find out all the data points that make up a spike is crucial to statistically analyze the area of spikes. In order to better identify current spikes and fit their shapes, our algorithm scans throughout all the data points in the whole current-time trace. As Eq. 2 puts, the top part of spike is determined from the first data point where the corresponding current value is greater than a threshold, to the point when the next point value is below the threshold. Since there have been no criteria to clearly define valid signals from background for NIE, we exploit the dispersion property of the data, and assume a large deviation from the mean, or in this case our baseline, would infer that a spike is likely in place. In practice, we use several standard deviations to quantify the dispersion (Pedone et al., 2009; Forstater et al., 2016). A detailed discussion of the procedure is shown in Supplementary Material *,*

$$S_{2}\left(t\right)=S_{0}\left(t\right),\;\;S_{0}\left(t\right)-S_{1}\left(t\right)\geq v$$ (2)

where *S*_*2*_(*t*) is the top part of spike value, and *v* is the threshold.

**FIGURE 2:**
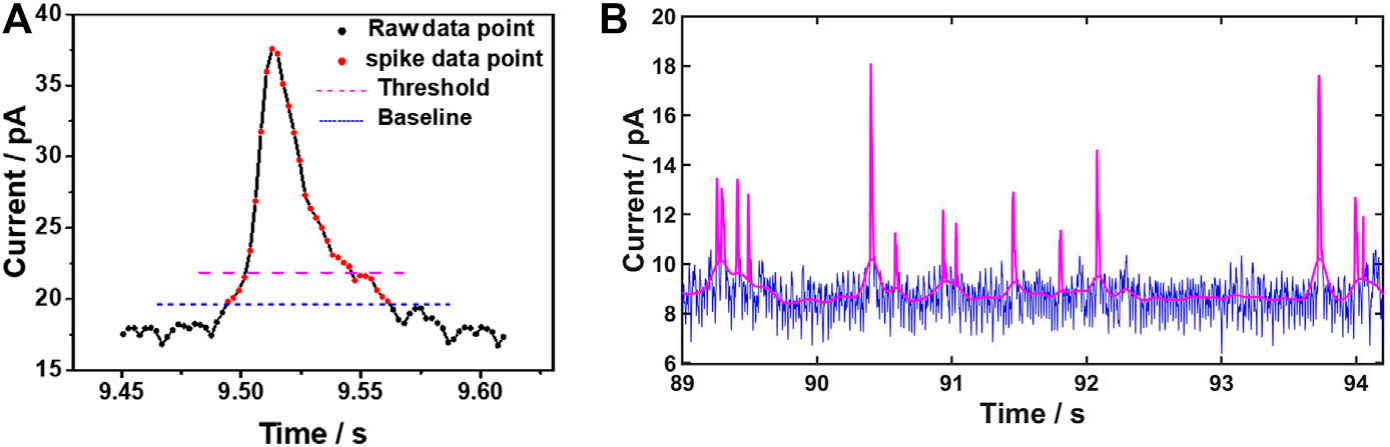
Baseline extraction and spike identification **(A)** Region of an identified single current spike. The blue and pink dashed lines represent the baseline and the defined threshold, respectively. The dots represent the experimental data points, with the discrete measurement points of a spike shown in red **(B)** Illustration of data processing on current-time trace using MATLAB. The blue line is the raw background noise and the pink line is a combination of baseline and spikes signals after computation.

As the defined threshold is commonly large enough to avoid the influence of the noise fluctuations, those data points belonging to the real spikes between the threshold and the baseline are very likely to be missed. The missing data points at the two edges of the spike will lead to a deviation from the original spike shape due to the incorrect recognition of the starting and the stopping point of the spike. As a consequence, a negative error would be induced in the spike integration, resulting in unreliable calculation of the charge transferred in single impact events. To restore the shape of the spike as much as possible, the data points near the threshold should be reevaluated. According to Figure 2A, any data points below the top part of the spike but above the baseline are treated as data points of the spike. Regardless the threshold, a reliable baseline can serve as an ultimate criterion to restore the spike shape. This process is implemented by MATLAB software as shown in Figure 2B.

**FIGURE 3:**
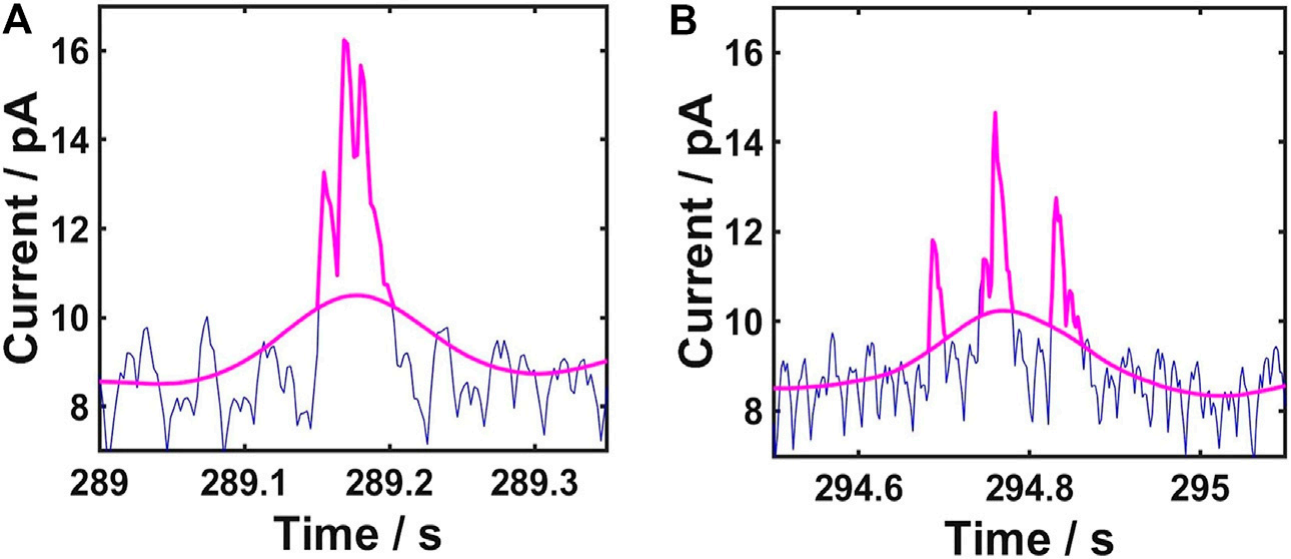
Rule to identify single and multiple AgNP impact **(A)** Troughs of a cluster of spikes above the baseline indicate that the spike clusters are from multiple impact events of a single AgNP **(B)** Troughs of a cluster of spikes below the baseline indicate that the spike clusters are individual spikes from multiple single AgNPs.

It was reported that single AgNPs might undergo a dynamic multiple impact events on a microelectrode surface, resulting in irregular spike clusters (Oja et al., 2017; Ustarroz et al., 2017; Ma et al., 2020). With regard to this, our method sets up a rule to deal with these spike clusters. As shown in Figure 3, if the troughs of a cluster of spikes are above the baseline, the spike clusters are considered to be resulted from multiple impact events of a single AgNP. If not, we treat them as individual spikes resulting from multiple single AgNPs.

### Spike Area Integration

Accurate recognition of all the spike points to preserve the original features of the spike shape is the key to calculate spike area accurately. To perform definite integration of the known data points that lacks a functional expression, trapezoidal numerical integration can be adopted (Eq. 3), where the spike points are integrated within the duration from the starting point to the stopping point,

$$\int_{t_{a}}^{t_{b}}S_{2}\left(t\right)\mathrm{dt}=\;\frac{t_{b}-t_{a}}{2N}\sum_{t=t_{a}}^{t=t_{b-\mathrm{\Delta T}}}\left(S_{2}\left(t\right)+\;S_{2}\left(t+\Delta T\right)\right)$$ (3)

where *t*_*a*_ and *t*_*b*_ are time coordinates of the starting point and the stopping point of a spike, *N* is the number of the spike points, and Δ*T* is the sampling interval. In this way, the individual spike area can be obtained, which is corresponding to the charge passed per oxidative spike.

### Automated Data Processing for AgNPs Oxidation in Impact Studies

**FIGURE 4:**
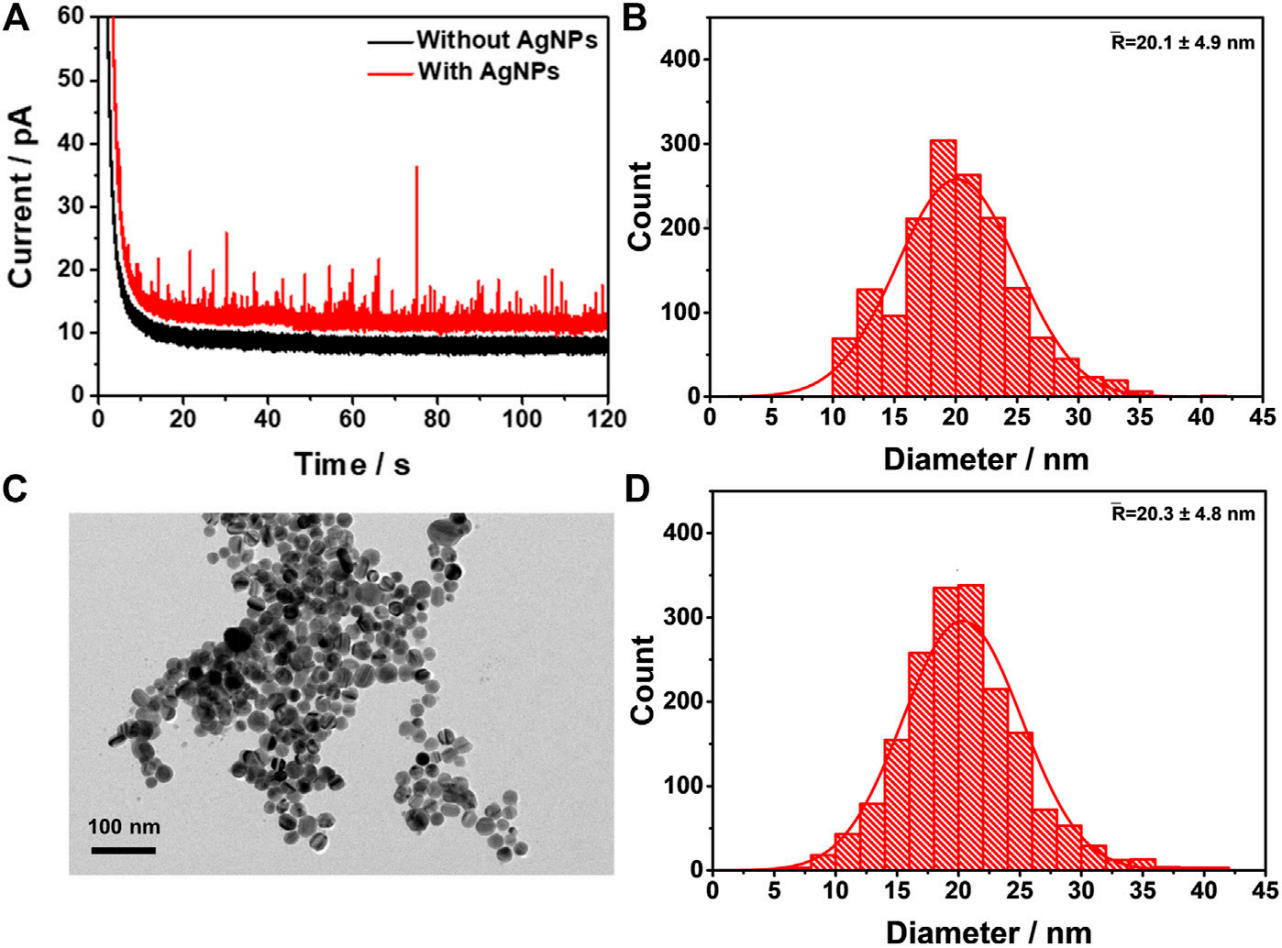
Verification of the spike detection algorithm **(A)** A representative chronoamperometric profile of AgNPs NIE. The black and red line indicate the absence and the presence of the AgNPs in solution, respectively **(B)** Histogram of the size distribution of AgNPs from the integrated charge processed by the algorithm **(C)** A representative TEM image of the AgNPs **(D)** Histogram of the size distribution of AgNPs derived from TEM determined by a Nano Measurer software.

To validate the aforementioned algorithm, it was applied to the automated data processing of the oxidation of AgNPs in nano-impact studies. The nano-impact experiments were carried out in a solution of 10 mM KCl containing 600 pM dispersed AgNPs, with a carbon fiber microelectrode potentiostatted under 0.6 V (SCE). In such experimental conditions, AgNPs were considered to be completely oxidized according to previous reports (Zhou et al., 2011; Ustarroz et al., 2017). The obtained chronoamperometric profile was shown in Figure 4A, where a large number of oxidative current spikes were recorded (red line), while no spikes were observed when AgNPs were absent in the electrolyte (black line), indicating that AgNPs impacts are the source of the oxidative spikes. Using Eq. 3, the charge passed per individual spike was obtained, which can then be correlated with the radius of the AgNPs assuming that the nanoparticles are spherical according to Eq. 4 (Zhou et al., 2011),

$$Q=\frac{4\mathrm{\pi n\rho F}r^{3}}{3A_{r}}$$ (4)

where *r* is the radius of the nanoparticle, *A*_*r*_ is the molar mass, *F* is the Faraday constant, *n* is the number of electrons transferred per atom in the nanoparticle, and *ρ* is the density of the nanoparticle.

The corresponding size distribution ofAgNPs was shown in Figure 4B, exhibiting a mean diameter of 20.1 ± 4.9 nm. Next, AgNPs were characterized by TEM (Figure 4C), which yields a size distribution of AgNPs (Figure 4D) determined by Nano Measurer software, with a mean diameter of 20.3 ± 4.8 nm. It is clear that the size from the automated data processing agrees very well with the TEM characterization, suggesting that our algorithm is able to reliably analyze the oxidative signals of AgNPs obtained in nano-impact experiments.

## Conclusion

Efficient and reliable data analysis in NIE is challenging due to the large data volume and the interference from the background noise. Here, we propose an automated data processing method named spike detection algorithm, including baseline extraction based on moving average filter, spike identification *via* the recognition of each of spike points by setting thresholds, and spike area integration using trapezoidal numerical integration. Using NIE of direct AgNPs oxidation as a model, the feasibility of the current algorithm is successfully verified, demonstrating that it is able to provide high level of accuracy and efficiency in processing NIE data and to push forward the current manual fitting into the stage of automatic data processing. This method may provide opportunities for applying NIE into high-throughput sensing applications.

## Data Availability

The raw data supporting the conclusions of this article will be made available by the authors, without undue reservation.

## References

[B1] Bartlett T. R. Sokolov S. V. Compton R. G. (2015). Electrochemical Nanoparticle Sizing via Nano-Impacts: How Large a Nanoparticle Can Be Measured? ChemistryOpen 4 (5), 600–605. 10.1002/open.201500061 26491639 PMC4608527

[B2] Batchelor-McAuley C. Ellison J. Tschulik K. Hurst P. L. Boldt R. Compton R. G. (2015). *In Situ* nanoparticle Sizing with Zeptomole Sensitivity. Analyst 140 (15), 5048–5054. 10.1039/C5AN00474H 26050623

[B3] Bunga Y. Kataky R. (2019). Silver Nanoparticle Impacts on Gold Electrode Surfaces in Flow-Injection Configuration. Sensors Actuators B: Chem. 290, 140–146. 10.1016/j.snb.2019.03.065

[B4] Chen Y. Wang D. Liu Y. Gao G. Zhi J. (2021). Redox Activity of Single Bacteria Revealed by Electrochemical Collision Technique. Biosens. Bioelectron. 176, 112914. 10.1016/j.bios.2020.112914 33353760

[B5] Cheng W. Compton R. G. (2016). Measuring the Content of a Single Liposome through Electrocatalytic Nanoimpact “Titrations”. ChemElectroChem. 3 (12), 2017–2020. 10.1002/celc.201600396

[B6] Cheng W. Batchelor-McAuley C. Compton R. G. (2014). Organic Nanoparticles: Mechanism of Electron Transfer to Indigo Nanoparticles. ChemElectroChem. 1 (4), 714–717. 10.1002/celc.201300233

[B7] Dasari R. Robinson D. A. Stevenson K. J. (2013). Ultrasensitive Electroanalytical Tool for Detecting, Sizing, and Evaluating the Catalytic Activity of Platinum Nanoparticles. J. Am. Chem. Soc. 135 (2), 570–573. 10.1021/ja310614x 23270578

[B8] Dick J. E. Renault C. Bard A. J. (2015). Observation of Single-Protein and DNA Macromolecule Collisions on Ultramicroelectrodes. J. Am. Chem. Soc. 137 (26), 8376–8379. 10.1021/jacs.5b04545 26108405

[B9] Dick J. E. (2016). Electrochemical Detection of Single Cancer and Healthy Cell Collisions on a Microelectrode. Chem. Commun. 52 (72), 10906–10909. 10.1039/C6CC04515D 27533129

[B10] Du M. Meng Y. Zhu G. Gao M. Hsu H.-Y. Liu F. (2020). Intrinsic Electrocatalytic Activity of a Single IrOx Nanoparticle towards Oxygen Evolution Reaction. Nanoscale 12 (43), 22014–22021. 10.1039/D0NR05780K 33140807

[B11] Dunevall J. Fathali H. Najafinobar N. Lovric J. Wigström J. Cans A.-S. (2015). Characterizing the Catecholamine Content of Single Mammalian Vesicles by Collision-Adsorption Events at an Electrode. J. Am. Chem. Soc. 137 (13), 4344–4346. 10.1021/ja512972f 25811247

[B12] Feng A. Cheng W. Compton R. G. (2016). Measuring the Oxygen Content of a Single Oil Droplet. Chem. Sci. 7 (10), 6458–6462. 10.1039/C6SC02357F 28451103 PMC5355955

[B13] Forstater J. H. Briggs K. Robertson J. W. F. Ettedgui J. Marie-Rose O. Vaz C. (2016). MOSAIC: A Modular Single-Molecule Analysis Interface for Decoding Multistate Nanopore Data. Anal. Chem. 88 (23), 11900–11907. 10.1021/acs.analchem.6b03725 27797501 PMC5516951

[B14] Gao G. Wang D. Brocenschi R. Zhi J. Mirkin M. V. (2018). Toward the Detection and Identification of Single Bacteria by Electrochemical Collision Technique. Anal. Chem. 90 (20), 12123–12130. 10.1021/acs.analchem.8b03043 30209941

[B15] Gooding J. J. (2016). Single Entity Electrochemistry Progresses to Cell Counting. Angew. Chem. Int. Ed. 55 (42), 12956–12958. 10.1002/anie.201606459 27531025

[B16] Gu Z. Ying Y.-L. Cao C. He P. Long Y.-T. (2015). Accurate Data Process for Nanopore Analysis. Anal. Chem. 87 (2), 907–913. 10.1021/ac5028758 25514172

[B17] Haddou B. Rees N. V. Compton R. G. (2012). Nanoparticle-electrode Impacts: the Oxidation of Copper Nanoparticles Has Slow Kinetics. Phys. Chem. Chem. Phys. 14 (39), 13612–13617. 10.1039/C2CP42585H 22965023

[B18] Kätelhön E. Tanner E. E. L. Batchelor-McAuley C. Compton R. G. (2016). Destructive Nano-Impacts: What Information Can Be Extracted from Spike Shapes?. Electrochimica Acta 199, 297–304. 10.1016/j.electacta.2016.02.031

[B19] Kanokkanchana K. Saw E. N. Tschulik K. (2018). Nano Impact Electrochemistry: Effects of Electronic Filtering on Peak Height, Duration and Area. ChemElectroChem. 5 (20), 3000–3005. 10.1002/celc.201800738

[B20] Kim B.-K. Kim J. Bard A. J. (2015). Electrochemistry of a Single Attoliter Emulsion Droplet in Collisions. J. Am. Chem. Soc. 137 (6), 2343–2349. 10.1021/ja512065n 25616104

[B21] Kirk K. A. Vasilescu A. Andreescu D. Senarathna D. Mondal S. Andreescu S. (2021). Collision-Based Electrochemical Detection of Lysozyme Aggregation. Anal. Chem. 93 (4), 2026–2037. 10.1021/acs.analchem.0c03578 33416307

[B22] Lebègue E. Barrière F. Bard A. J. (2020). Lipid Membrane Permeability of Synthetic Redox DMPC Liposomes Investigated by Single Electrochemical Collisions. Anal. Chem. 92 (3), 2401–2408. 10.1021/acs.analchem.9b02809 31916438

[B23] Lee J. Y. Kim B.-K. Kang M. Park J. H. (2016). Label-Free Detection of Single Living Bacteria via Electrochemical Collision Event. Sci. Rep. 6 (1), 30022. 10.1038/srep30022 27435527 PMC4951717

[B24] Lee J. Gerelkhuu Z. Song J. Seol K. H. Kim B.-K. Chang J. (2019). Stochastic Electrochemical Cytometry of Human Platelets via a Particle Collision Approach. ACS Sens. 4 (12), 3248–3256. 10.1021/acssensors.9b01773 31680513

[B25] Lee J. Kang Y. Chang J. Song J. Kim B.-K. (2020). Determination of Serotonin Concentration in Single Human Platelets through Single-Entity Electrochemistry. ACS Sens. 5 (7), 1943–1948. 10.1021/acssensors.0c00267 32498511

[B26] Li M. Ge Z. Zhang S. He P. Gu Y. Qi L. (2018). Electrocatalytic Reduction of Hydrogen Peroxide by Pd−Ag Nanoparticles Based on the Collisional Approach. ChemElectroChem. 5 (20), 3021–3027. 10.1002/celc.201801249

[B27] Lin C. Kätelhön E. Sepunaru L. Compton R. G. (2017). Understanding Single Enzyme Activity via the Nano-Impact Technique. Chem. Sci. 8 (9), 6423–6432. 10.1039/C7SC02084H 29163928 PMC5632796

[B28] Liu Y. Xu C. Yu P. Chen X. Wang J. Mao L. (2018). Counting and Sizing of Single Vesicles/Liposomes by Electrochemical Events. ChemElectroChem. 5 (20), 2954–2962. 10.1002/celc.201800616

[B29] Ma W. Ma H. Chen J.-F. Peng Y.-Y. Yang Z.-Y. Wang H.-F. (2017). Tracking Motion Trajectories of Individual Nanoparticles Using Time-Resolved Current Traces. Chem. Sci. 8 (3), 1854–1861. 10.1039/C6SC04582K 28553475 PMC5424808

[B30] Ma H. Chen J.-F. Wang H.-F. Hu P.-J. Ma W. Long Y.-T. (2020). Exploring Dynamic Interactions of Single Nanoparticles at Interfaces for Surface-Confined Electrochemical Behavior and Size Measurement. Nat. Commun. 11 (1), 2307. 10.1038/s41467-020-16149-0 32385284 PMC7210955

[B31] Ngamchuea K. Clark R. O. D. Sokolov S. V. Young N. P. Batchelor-McAuley C. Compton R. G. (2017). Single Oxidative Collision Events of Silver Nanoparticles: Understanding the Rate-Determining Chemistry. Chem. Eur. J. 23 (63), 16085–16096. 10.1002/chem.201703591 28922508

[B32] Oja S. M. Robinson D. A. Vitti N. J. Edwards M. A. Liu Y. White H. S. (2017). Observation of Multipeak Collision Behavior during the Electro-Oxidation of Single Ag Nanoparticles. J. Am. Chem. Soc. 139 (2), 708–718. 10.1021/jacs.6b11143 27936665

[B33] Patrice F. T. Qiu K. Zhao L.-J. Kouadio Fodjo E. Li D.-W. Long Y.-T. (2018). Electrocatalytic Oxidation of Tris(2-Carboxyethyl)phosphine at Pyrroloquinoline Quinone Modified Carbon Nanotube through Single Nanoparticle Collision. Anal. Chem. 90 (10), 6059–6063. 10.1021/acs.analchem.7b05405 29701064

[B34] Pedone D. Firnkes M. Rant U. (2009). Data Analysis of Translocation Events in Nanopore Experiments. Anal. Chem. 81 (23), 9689–9694. 10.1021/ac901877z 19877660

[B35] Pendergast A. D. Deng Z. Maroun F. Renault C. Dick J. E. (2021). Revealing Dynamic Rotation of Single Graphene Nanoplatelets on Electrified Microinterfaces. ACS Nano 15 (1), 1250–1258. 10.1021/acsnano.0c08406 33325229

[B36] Peng Y. Y. Guo D. Ma W. Long Y. T. (2018). Intrinsic Electrocatalytic Activity of Gold Nanoparticles Measured by Single Entity Electrochemistry. ChemElectroChem. 5 (20), 2982–2985. 10.1002/celc.201801065

[B37] Rees N. V. Zhou Y.-G. Compton R. G. (2011). The Aggregation of Silver Nanoparticles in Aqueous Solution Investigated via Anodic Particle Coulometry. ChemPhysChem. 12 (9), 1645–1647. 10.1002/cphc.201100207 21560222

[B38] Sepunaru L. Tschulik K. Batchelor-McAuley C. Gavish R. Compton R. G. (2015). Electrochemical Detection of Single *E. coli* Bacteria Labeled with Silver Nanoparticles. Biomater. Sci. 3 (6), 816–820. 10.1039/C5BM00114E 26221841

[B39] Sepunaru L. Plowman B. J. Sokolov S. V. Young N. P. Compton R. G. (2016). Rapid Electrochemical Detection of Single Influenza Viruses Tagged with Silver Nanoparticles. Chem. Sci. 7 (6), 3892–3899. 10.1039/C6SC00412A 30155033 PMC6013776

[B40] Sokolov S. V. Tschulik K. Batchelor-McAuley C. Jurkschat K. Compton R. G. (2015). Reversible or Not? Distinguishing Agglomeration and Aggregation at the Nanoscale. Anal. Chem. 87 (19), 10033–10039. 10.1021/acs.analchem.5b02639 26352558

[B41] Sokolov S. V. Eloul S. Kätelhön E. Batchelor-McAuley C. Compton R. G. (2017). Electrode-particle Impacts: a Users Guide. Phys. Chem. Chem. Phys. 19 (1), 28–43. 10.1039/C6CP07788A 27918031

[B42] Stuart E. J. E. Zhou Y.-G. Rees N. V. Compton R. G. (2012). Determining Unknown Concentrations of Nanoparticles: the Particle-Impact Electrochemistry of Nickel and Silver. RSC Adv. 2 (17), 6879–6884. 10.1039/C2RA20628E

[B43] Tschulik K. Haddou B. Omanović D. Rees N. V. Compton R. G. (2013). Coulometric Sizing of Nanoparticles: Cathodic and Anodic Impact Experiments Open Two Independent Routes to Electrochemical Sizing of Fe3O4 Nanoparticles. Nano Res. 6 (11), 836–841. 10.1007/s12274-013-0361-3

[B44] Ustarroz J. Kang M. Bullions E. Unwin P. R. (2017). Impact and Oxidation of Single Silver Nanoparticles at Electrode Surfaces: One Shot versus Multiple Events. Chem. Sci. 8 (3), 1841–1853. 10.1039/C6SC04483B 28553474 PMC5424807

[B45] Wan Y. Guo Z. Jiang X. Fang K. Lu X. Zhang Y. (2013). Quasi-spherical Silver Nanoparticles: Aqueous Synthesis and Size Control by the Seed-Mediated Lee-Meisel Method. J. Colloid Interf. Sci. 394, 263–268. 10.1016/j.jcis.2012.12.037 23332939

[B46] Wang H. Yang C. Tang H. Li Y. (2021). Stochastic Collision Electrochemistry from Single G-Quadruplex/Hemin: Electrochemical Amplification and MicroRNA Sensing. Anal. Chem. 93 (10), 4593–4600. 10.1021/acs.analchem.0c05055 33660976

[B47] Xiang Z.-P. Deng H.-Q. Peljo P. Fu Z.-Y. Wang S.-L. Mandler D. (2018). Electrochemical Dynamics of a Single Platinum Nanoparticle Collision Event for the Hydrogen Evolution Reaction. Angew. Chem. Int. Ed. 57 (13), 3464–3468. 10.1002/anie.201712454 29377523

[B48] Xu W. Zhou Y. Ji X. (2018). Lithium-Ion-Transfer Kinetics of Single LiFePO4 Particles. J. Phys. Chem. Lett. 9 (17), 4976–4980. 10.1021/acs.jpclett.8b02315 30114916

[B49] Xu C.-Y. Yu R.-J. Ni X. Xu S.-W. Fu X.-X. Wan Y.-J. (2021). An Envelope Algorithm for Single Nanoparticles Collision Electrochemistry. Chin. J. Chem. 39 (7), 1936–1940. 10.1002/cjoc.202100079.

[B50] Zampardi G. Batchelor-McAuley C. Kätelhön E. Compton R. G. (2017). Lithium-Ion-Transfer Kinetics of Single LiMn2 O4 Particles. Angew. Chem. Int. Ed. 56 (2), 641–644. 10.1002/anie.201610485 27921361

[B51] Zhang J.-H. Shen Q. Zhou Y.-G. (2021). Quantification of Tumor Protein Biomarkers from Lung Patient Serum Using Nanoimpact Electrochemistry. ACS Sens 6 (6), 2320–2329. 10.1021/acssensors.1c00361 34033456

[B52] Zhou Y.-G. Rees N. V. Compton R. G. (2011). The Electrochemical Detection and Characterization of Silver Nanoparticles in Aqueous Solution. Angew. Chem. Int. Ed. 50 (18), 4219–4221. 10.1002/anie.201100885 21472836

[B53] Zhou Y.-G. Haddou B. Rees N. V. Compton R. G. (2012a). The Charge Transfer Kinetics of the Oxidation of Silver and Nickel Nanoparticles via Particle-Electrode Impact Electrochemistry. Phys. Chem. Chem. Phys. 14 (41), 14354–14357. 10.1039/C2CP42940C 23007231

[B54] Zhou Y.-G. Rees N. V. Pillay J. Tshikhudo R. Vilakazi S. Compton R. G. (2012b). Gold Nanoparticles Show Electroactivity: Counting and Sorting Nanoparticles upon Impact with Electrodes. Chem. Commun. 48 (2), 224–226. 10.1039/C1CC16407D 22086114

[B55] Zhou Y.-G. Rees N. V. Compton R. G. (2013). Electrochemistry of Nickel Nanoparticles Is Controlled by Surface Oxide Layers. Phys. Chem. Chem. Phys. 15 (3), 761–763. 10.1039/C2CP43618C 23207499

