# Supplementary material for: Rapid and Accurate Data Processing for Silver Nanoparticle Oxidation in Nano-Impact Electrochemistry
Source: Front Chem. 2021 Jul 2;9:718000. doi: 10.3389/fchem.2021.718000 (PMC8350773; doi:10.3389/fchem.2021.718000)
Supplement: Supplementary file 1 [file DataSheet1.docx]

Supplementary Material

Threshold selection

Our spike detection algorithm rests upon the observation that background noise, which is due to the electrochemical measurement setup, fluctuates around a stable level whether the spikes are present or not, as shown in Supplementary Figure 1. A large enough deviation from the mean background noise level, also known as the baseline, would indicate a spike is in place. Besides, the spike always results in a large positive current change, hence, a cutoff *threshold* is set as the baseline plus a certain amount of data deviation to separate out signals from the noise. In this case, we used standard deviation (σ) as the measure of data dispersion, and then carefully chose the desired threshold as 5σ away from the mean (ΔI_mean_), since a smaller deviation level would result in more questionable spikes (*i.e.*, more false positives, or low precision rate) and a larger value would result in less spike signals (*i.e.*, more false negatives, or low recall rate).


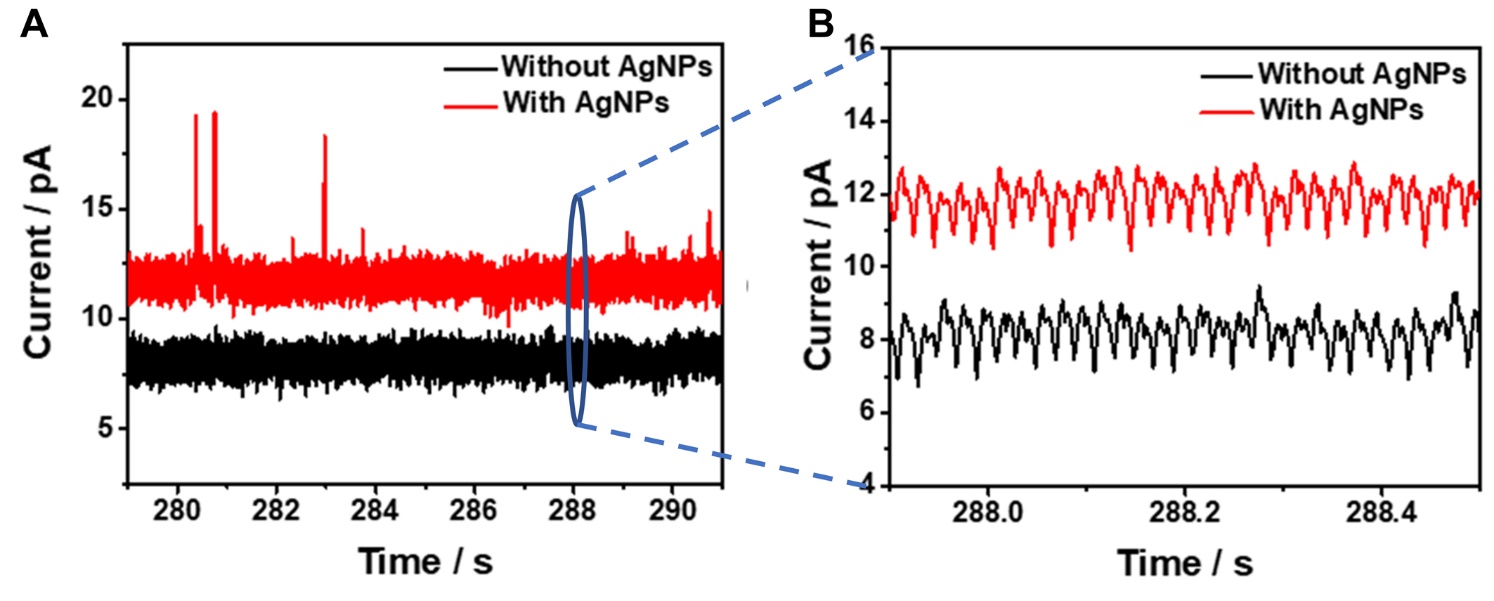


**Supplementary Figure 1.** Background noise with and without the presence of AgNPs in solution. (A) A representative chronoamperometric profile of AgNPs NIE. (B) Details of the current fluctuations of the background noise. The black and red line indicate the absence and the presence of the AgNPs, respectively.

From those detected spikes, we may further infer the size of the underlying nanoparticles. We also ran a statistical calculation of those size distributions, shown in Supplementary Figure 2, where our computational result demonstrates a good agreement with that from the TEM experiments, which justifies our 5σ deviation rule. Note that the probability of observing a nanoparticle is taken to be proportional to its diffusion coefficient so that smaller nanoparticles may conduct more frequent collision than the big ones. Accordingly, the sizing was corrected via weighting each nanoparticle by nanoparticle radius (r) (Little et al., 2018). The new size distributions obtained under different thresholds after weighting were shown Supplementary Figure 3. Compare with TEM analysis, 5σ remains the optimal threshold.


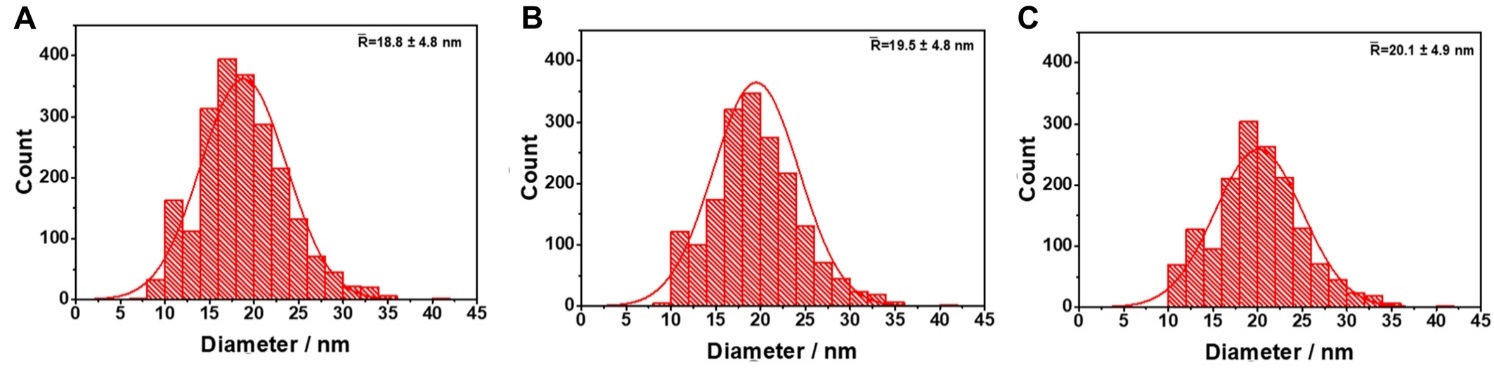
**Supplementary Figure 2.** Influences of threshold selection on the histogram of the size distribution of AgNPs. Size distribution obtained from the integrated charge with a threshold setting of (A) ΔI_mean_ + 3σ, (B) Δ I_mean_ + 4σ, and (C) Δ I_mean_ + 5σ.


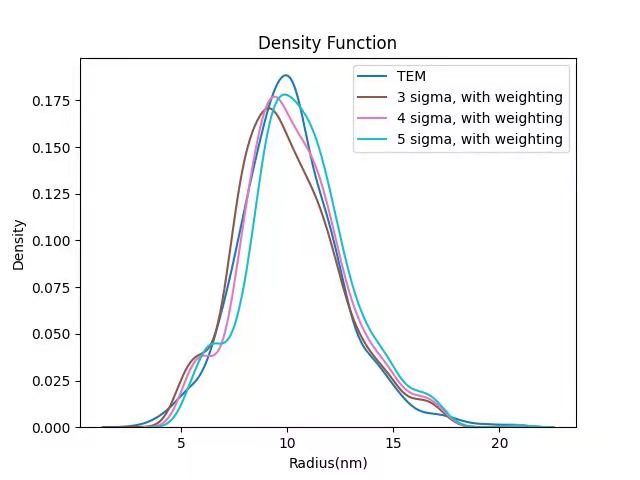


**Supplementary Figure 3.** The radius distributions of AgNPs after weighting. The blue line was obtained from the TEM images. The brown, pink and cyan lines were obtained from the integrated charge under different thresholds (ΔI_mean_ + 3σ, Δ I_mean_ + 4σ, and Δ I_mean_ + 5σ).

**References：**

Little, C.A., Xie, R., Batchelor-McAuley, C., Kätelhön, E., Li, X., Young, N.P., et al. (2018). A quantitative methodology for the study of particle–electrode impacts. Physical Chemistry Chemical Physics 20(19), 13537-13546. doi: 10.1039/C8CP01561A.
